# Endogenous Retroviruses: Thierry Heidmann wins the 2009 *Retrovirology *prize

**DOI:** 10.1186/1742-4690-6-108

**Published:** 2009-12-01

**Authors:** Ali Saib, Monsef Benkirane

**Affiliations:** 1 Conservatoire National des Arts et Metiers, CNRS UMR7212/INSERM U944, Paris, France; 2 Institut de Genetique Humaine, CNRS UPR1142, Montpellier, France

## Abstract

Thierry Heidmann wins the 2009 *Retrovirology *prize.

## 

In 2005, thanks to the generosity of the Ming K. Jeang Foundation, the *Retrovirology *prize was inaugurated. A goal of the *Retrovirology Prize *is to identify an outstanding mid-career scientist who is close to the peak of his/her productivity and who is expected to have many future years of high achievement. Previous winners of the *Retrovirology Prize *include Stephen Goff [1], Joseph Sodroski [2], Karen Beemon [3] and Ben Berkhout [4].

For 2009, the Editors of *Retrovirology *have selected Thierry Heidmann (Figure 1) as the recipient of the *Retrovirology Prize*.

**Figure 1:**
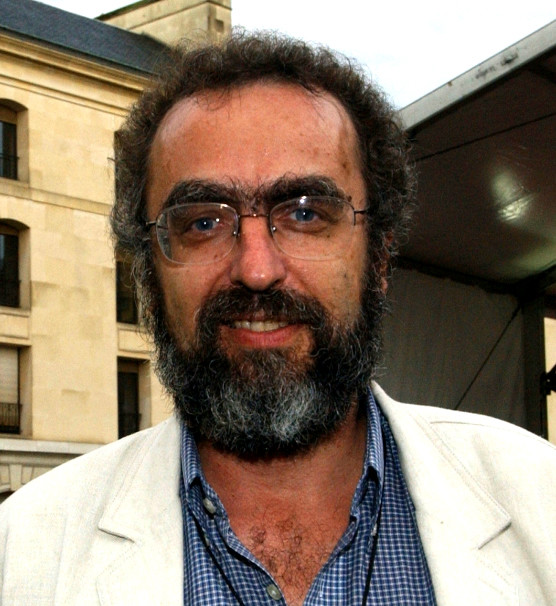
**Thierry Heidmann**.

TH heads a CNRS laboratory at Université Paris-Sud and Institut Gustave Roussy in Villejuif, in the southern suburbs of Paris. With a strong background in physics and mathematics, he was interested in the early 1980's in linking these disciplines of science with biology, more precisely with neurobiology and the science of complex neuronal networks. To fulfil his aspiration, Thierry entered Jean-Pierre Changeux's lab at the Pasteur Institute to start a PhD on the newly discovered acetylcholine receptor. This exciting experience led Thierry to turn to more applied science, following a profound will to slowly but surely shift from studying the physical sciences to studying the biological sciences! After his PhD, Thierry focused on transposable elements and retrotransposons. The question he asked at that time was simple: how to be able to monitor the transposition of these mobile elements in a mammal where genetic approaches to detect transposition were both poorly efficient and time consuming. He then decided to devise a system that could detect retrotransposition of any element whose mobilization includes a reverse transcription step, no matter where the reverse transcribed, transposed element would target and integrate. This search resulted in 1988 in the generation of the first indicator gene for retrotransposition. This was his first step in the field, but a decisive one. Since then, his laboratory has focused on three complementary directions: the regulation of retroelements' activities, the search for functional endogenous retroviruses, and their role in physiological and pathological conditions.

Professor Ali Saib, a member of the editorial board of *Retrovirology *http://www.Retrovirology.com asked him a series of questions on these elements and about his views on several issues raised by his investigations.

## AS: Before becoming interested in endogenous retroviruses, you studied neurobiology. What did you work on?

**TH**: In fact, I followed a rather atypical path. I attended the "École Normale Supérieure" in Paris where I learned physics and mathematics. Initially, I was thinking about astrophysics, but I rapidly became interested in the multiple attempts, that at that time were already very popular, to make a link between these domains of science and that of biology, and more precisely with neurobiology and the science of complex neuronal networks. Therefore, I rapidly looked for laboratories dedicated to biology that had a special interest in these aspects of science, and I was very happy to find a very open-minded scientist, who was able both to think about receptor-ligand interactions at the molecular level and to build models for neuronal networks and integrated thinking processes, in the person of Jean-Pierre Changeux, at the Pasteur Institute. So in the course of the ENS program, I entered Jean-Pierre Changeux's lab and started PhD work on the newly discovered and still very mysterious acetylcholine receptor. My approach was initially that of a physicist, and I managed to identify the rapid allosteric transitions that proved to be associated with the binding of acetylcholine to its receptor, and resulting in the opening of the ion channel. A step further was achieved with the characterization of the receptor-associated channel, which proved to be made of the central space generated by the pentameric arrangement of the receptor, and that we managed to label in its closed and open states by constructing an apparatus allowing both the rapid addition of acetylcholine to the receptor, that of a non-competitive blocker of the ion channel and the real-time labeling of the latter by millisecond UV irradiation of the complex. A comprehensive allosteric model for neurotransmitter action as initially postulated by Jean-Pierre Changeux was therefore confirmed. These biochemical and physico-chemical studies also led us to propose a model for the regulation of synaptic efficacy, based on the unexpected observation that the receptor could exist under two conformations in reversible equilibrium. So that was my first experience in Biology.

## AS: In choosing a new research direction, why did you focus on endogenous retroviruses and not on HIV, which was much more in the lime light. It was a difficult choice in France, or even internationally, where this topic wasn't (and still isn't) very developed?

**TH**: After my PhD on this rather theoretical work in molecular neurobiology, I decided to turn to more applied science, following a profound willingness to slowly but surely shift from physical to medical sciences and   cope with human diseases. So I began to think about tumors, in relation with transposable elements, retrotransposons, and retroviruses, being aware that in several species where genetics had been a powerful tool such as Drosophila and mice, it was clear that a large fraction of the observed mutations (eye pigmentation in Drosophila, fur color in mice) were associated with insertional mutagenesis, mediated by mobile elements. At that time, a very illuminating paper had just been published by Boeke et al. in *Cell*, showing that the Ty element in yeast was a retrotransposon, i.e. an element that requires a reverse transcription step to replicate, in a strictly intracellular process, thus incredibly resembling the replicative cycle of retroviruses, although the latter have an extracellular life cycle. A minireview by Baltimore also in *Cell *had very nicely stressed these similarities. So it could be envisioned that what oncogenic retroviruses can do simply by stochastic insertion into the host genome, maybe endogenous elements could also do it, provided that they are transcriptionally active. And the first question that I asked was simple: how to monitor the mobility of these elements in a mammal where genetic approaches to detect transposition were both inefficient and long-drawn-out? Indeed, only a fraction of the insertional events are expected to result in a mutation associated with a detectable phenotypic trait, and therefore I decided to design a system that could detect retrotransposition of any element mobilized by a process involving a reverse transcription step, whatever the target and integration site of the reverse transcribed, transposed element. This search led in 1988 to the generation of the first « indicator gene for retrotransposition » [5] a device which included a marker gene (neomycin resistance or lacZ) interrupted by an intronic sequence that inactivates the marker gene in its initial form and which is eliminated in the course of processing of the transitory « RNA » intermediate of the retrotransposon, with the transposed element hence expressing the marker gene. This indicator gene in its various forms is now widely used by all labs working on retroelements and actually proved to be extremely powerful, both *in vitro *and *in vivo*, to characterize the mechanism of transposition of retroelements, and to study the regulation mechanisms that nature has imposed on them so that they do not wreak genomic havoc. That was the way I entered the retrovirology field!

## AS: Was it difficult to make such a shift?

**TH**: In fact not at all! I have to say that I could take advantage of the French system for academic research, which gave me the opportunity to propose a new research program just after my PhD. Although it was not very classical, I did not make a « true » post-doc but I started immediately my new project. At the beginning, I was doing molecular biology in Jean-Pierre Changeux's lab, who kindly let me start on a totally non-neurobiological project (!), and I was doing cell culture and retrovirology with Jean-François Nicolas in François Jacob's lab, that was located just one floor below. This period lasted a few months, and it was really a wonderful time; and then I searched for a lab in the field and I rather naturally turn to Jean-Bernard LePecq who was at the time director of a CNRS unit at the Institut Gustave Roussy, and who provided me with a small lab in the newly constructed Research Department. The Institut Gustave Roussy is devoted to cancer research, and was interested in my proposed research program, that actually they have always supported, together with the CNRS all along these years. So I was not at that time focused on HIV, and the scientific authorities from whom I was dependent were in fact interested in tumor biology. Maybe I should add that my atypical formation, my strong determination to explore the new field of retroelements and endogenous retroviruses, and the relative freedom allowed within our academic research system, were sufficient arguments to let me go.

## AS: What are the most important advances to which you contributed?

**TH**: Actually, the first element that I investigated for its possible retrotransposition was a simple retrovirus, the mouse leukemia virus (MLV), whose *env *gene we deleted and replaced with the indicator gene for retrotransposition. And we could demonstrate that it was able to retrotranspose intracellularly, thus clearly showing the very close link that exists between retroviruses and retrotransposons. This « principal » paper also depicted a clear-cut method for quantitative measurement of retrotransposition frequency of a given element in a given cell line (as straightforward as for bacteria using the Luria & Delbruck or the Newcombe methods). This initial demonstration was followed by several others, including the demonstration and follow-up of the retrotransposition of a mouse IAP sequence that we had marked and which proved to retrotranspose very efficiently in a tumor cell line [6], and the demonstration that the LINE elements, i.e. non-LTR retrotransposons, also retrotranspose, as do Alu sequences, the human prototypic representative member of the non-coding SINEs. For the LINEs, we did not hesitate to turn to Drosophila where a functional LINE element -the I element- had been identified and cloned by Bucheton's group, and which could be mobilized by appropriate crosses between Drosophila contaminated by this element and Drosophila still free of the element. This element was marked by a neo^R^-containing indicator gene for retrotransposition; the element was introduced by transgenesis into Drosophila; and retrotransposition was followed by direct selection of the offspring by introducing the selecting drug directly into the food on which the larva thrived. And remarkably, some Drosophila were found to perfectly survive on this otherwise « lethal » food; the DNA of these survivors was analyzed, and indeed a transposed copy of the transgene was in each case identified, with the intron precisely spliced out, thus demonstrating unambiguously retrotransposition of LINEs, that had taken place in the mother germline. This work was extended about 5 years later by Kazazian's group working on human LINEs, using a functional LINE that they had identified and cloned from a patient where such an element had recently transposed. LINE were also demonstrated by our group to be involved in the generation of the so-called processed pseudogenes: these are in general non-functional copies of *bona fide *genes, but which lack introns, end with a polyA stretch, and lack a promoter, thus suggesting that they are most probably generated by a reverse transcription process, from a gene transcript. Again, thanks to the indicator gene of retrotransposition that we used to mark classical genes, we could reconstitute pseudogene formation *in vitro*, and further demonstrate that LINEs were carrying out this process very efficiently [7]. Not only LINEs were able to take charge of a gene transcript, but we also showed that they were responsible for the mobilization of SINE elements. These short and highly abundant elements of the human and mouse genome (more than a million copies) are mobile but non-coding, and are synthesized as a highly structured transcript by RNA Polymerase III. The latter property led us to devise a new indicator gene, in which the initial intronic sequence was replaced by a self-splicing intron, that was taken from Tetrahymena: this new indicator gene did not impose the RNA Polymerase II-dependent spliceosome pathway to be used by the marked retrotransposon, and indeed allowed a clear demonstration -and follow-up- of the retrotransposition of both the human Alu sequence, and the mouse B1 and B2 SINE, as well as the identification of the structural features of the RNA intermediate responsible for the incredibly efficient retrotransposition of these elements.

## AS: And what about ERVs?

**TH**: Our search for endogenous retroviruses (ERVs) started well ahead of the time when genomes were sequenced, and was based on the old observation -by electron microscopists- that virus-like particles can be observed in the placenta and in some tumors. Accordingly, we first searched for retrovirus-like sequences that might be expressed, by using the classical tools of molecular biology: RNA from freshly recovered placenta were assayed for the presence of *pol*-containing sequences, using RT-PCR with degenerate primers matching the highly conserved RT domains in the *pol *gene. Doing so, a class of endogenous retroviruses that were named HERV-L was identified, which proved to be a very ancient acquisition of animal genomes, before the radiation of mammals, about 100 million years (My) ago. These elements were found to be still active in the mouse where they had been subjected to an amplification burst after the divergence between mouse and rat, and to be responsible for the formation of the epsilon particles that had been identified in 2-cell embryos by electron microscopists 40 years ago and had remained orphan since then. In a way we made a sort of viral archeology, over million years of evolution.

Thanks to the progress in the sequencing of both the human and mouse genomes, it became rapidly possible to have a clear-cut view of the extent and impact of ERVs: they occupy as much as 8% of these genomes, with significant differences between mice and humans. In the mouse, an extensive characterization of the various families of ERVs led to the identification of several families among which we managed to identify fully functional copies, still able to replicate, with in some cases the surprising evidence that the number of remaining functional copies was as low as unity, thus suggesting that some ERV families can simply die out. A similar conclusion was actually derived from our ERV-L study, showing that survival of an ERV family is actually dependent on the subtle balance between replication efficiency and genetic drift, with the ability of a given element to make multiple functional copies of itself being the sole parameter responsible for its survival, independently of any selective pressure. Interestingly, we identified a specific feature of the very successful mouse ERVs, and especially of the IAP and MusD elements -which are responsible for the majority of the mutations by insertion identified in the mouse: these ERVs have acquired a strictly intracellular life cycle, at variance with their retroviral « progenitor ». And we proposed that this physical constraint, by preventing the dilution of the viral particles in the extracellular medium, favors the immediate integration of the replicating element within the producing cell genome. Interestingly, the natural history of this evolutionary process, that we named « intracellularization », could be reconstituted at the molecular level, and the nature of the viral progenitor of these elements even could be determined. For both elements we could demonstrate that intracellularization was simply due to mutations in the N-terminal domain of gag, which led to a change in the addressing of the viral particle from the cell membrane to either the cytoplasm or the membrane of the endoplasmic reticulum. This intracellularization resulted in degeneration of the *env *gene, with an envelope protein becoming useless for a particle no longer exiting the cell. In one case, that of the IAP sequences, a systematic search within the mouse genome led to the unambiguous identification of the viral progenitor of these intracellularized particles, with the identification of a functional proviral copy -named IAP-E- that proved to be able to generate fully infectious viral particles, budding at the cell membrane, and that could be converted by introducing the appropriate sequence at its gag N-terminus into a strictly intracellular ERV, indistinguishable from a *bona fide *IAP particle, being even able to efficiently undergo retrotransposition. Again, with this specific example, we could demonstrate the very intimate link between infectious retroviruses and retroelements, with the passage from one to the other, which had taken place over million years of evolution, being recapitulated in the laboratory.

## AS: What about the Human ERVs?

**TH**: In the case of human ERVs, the situation proved to be slightly different, since analysis of the human genome revealed that most -if not all- the proviral copies that can be identified are most probably defective for autonomous replication, their *gag*, *pol *and *env *genes being disrupted by stop codons, deletions and insertions. Yet, there is one exception, that of the HERV-K family of endogenous retroviruses, which displays human specific copies not found in the chimpanzee, from which we could « resurrect », for the first time, an infectious retrovirus -that we named Phoenix- by constructing a proviral genome based on a consensus sequence derived from the identified copies. A similarly active copy could also be obtained by recombination of two proviral copies, thus strongly suggesting that fully functional copies could possibly still be present in some individuals. Yet, such alleles remain to be found, and are actually searched for by several groups.

## AS: So many retroelements, but what about genome stability?

**TH**: You are right, retroelements occupy as much as 45% of the human or mouse genome, with 8% being ERVs. So in parallel, during these years, our laboratory -as well as others-has investigated the important issue of the regulation of the activity of retroelements. To be relevant, we carried out such studies essentially *in vivo*, using appropriate animal models (Drosophila and mice) and transgenesis. Consistent with the expected deleterious effects of insertional mutagenesis, a series of studies provided evidence for highly restricted activity of retroelements, and involvement of essential regulatory processes to « tame » these elements. Among the most salient outcomes from our laboratory, I would like to cite the *in vivo *demonstration of the restriction of the expression of IAP endogenous retroviruses to the male germline, using lacZ-marked elements introduced by transgenesis, the involvement of CpG-methylation in somatic restriction, and the evidence for somatic demethylation and induction of expression under specific conditions. In Drosophila, we could demonstrate -via the introduction by transgenesis of small non-coding fragments of the LINE I element- that I element activity is restricted by an « homology-dependent gene silencing » process, requiring transcripts from the introduced I fragments for mediating inhibition by RNA interference [8]. And finally, in collaboration with Olivier Schwartz at the Pasteur Institute, we could demonstrate the primary role of cellular APOBEC proteins in the taming of mammalian endogenous retroviruses, with the effects of APOBEC expression being demonstrated *ex vivo*, as well as *in vivo *through the analysis of the traces left in the course of evolution by APOBEC enzymes on the series of endogenous retroviruses that can be found in the mouse and human genome. Again these last results illustrate the strong similarities between ERVs and infectious RVs, with the APOBEC proteins having been initially discovered via their inhibitory effects on infectious retroviruses, but finally having most probably been selected million years ago to prevent genome invasion by retroelements.

## AS: ERVs could play a role not only via their mobility but also via expression of their proper genes; what about their *env *gene on which you have now focused most of your research?

**TH**: We rapidly focused on the retroviral envelope proteins, and made a systematic search for full length, intact *env *genes within the human genome. This search resulted in the identification of a rather low number of elements, with only 18 genes identified. Among them, 2 proved to be very important, as they are able to mediate cell-cell fusion *ex vivo*, are specifically expressed in the placenta, and are conserved in primate evolution. Syncytin-1 was discovered by two groups, one in the US and one in France, and syncytin-2 by our group. It became rapidly clear that these retroviral envelopes have been co-opted by their host -more than 25 My ago- for a physiological function in relation with the formation of the syncytiotrophoblast layer at the materno-fetal interface. Our laboratory has been involved in the characterization of syncytin-2, the oldest syncytin found in all simians, with the identification of its cognate receptor and evidence for its possible involvement in the « in-fusion » of the mononucleated cytotrophoblasts into the syncytiotrophoblast. Yet, a definitive demonstration of the role of syncytins in mammalian placentation was only recently obtained, via the mouse model and the generation of knockout animals. To do so, we first searched for syncytin genes in the mouse genome, and found two such genes that we named syncytin-A and -B, which proved to be divergent from the human syncytins and to correspond to totally independent « retroviral gene captures » by a rodent ancestor. These two genes possess all the characteristic features of *bona fide *syncytins, as they can mediate cell-cell fusion *ex vivo*, they are specifically expressed in the placenta at the maternal-fetal interface, and have been conserved over > 20 My of evolution of rodents. Then, knockout mice for one of these genes very simply provided evidence that syncytins are indeed absolutely required for placentation, with evidence for lack of syncytiotrophoblast formation, resulting in embryo death at mid-gestation [9]. Work is now in progress to determine whether syncytins can be found in all placental mammals (and a series of preliminary data are indicative that it is indeed the case), thus supporting the working hypothesis that placental mammals may have emerged via the capture of a primitive retroviral envelope, possibly replaced in the course of evolution by newly acquired and diverse envelopes such as those that can be observed today.

## AS: This is definite evidence for a positive effect of ERVs, but what about negative effects?

TH: One important outcome of the detailed study of retroviral envelopes from both exogenous and endogenous retroviruses was the demonstration that they possess an immunosuppressive activity that can be revealed in an *in vivo *assay based on the inhibition of tumor rejection by the mouse immune system. Actually, we showed in a « principal » paper [10] that tumor cells engrafted into immunocompetent mice are no longer rejected if they have been previously transduced by an expression vector for a retroviral envelope. In this paper, we further suggested that this property could be of importance for two major processes: in viremia for *bona fide *infectious retroviruses, where immunosuppression by the *env *gene could be essential for viral propagation in an immunocompetent animal, and in tumors where endogenous retroviruses are currently activated, and where the expressed endogenous envelopes could participate in tumor progression by inhibiting immunosurveillance. Consistent with the second hypothesis, we could unambiguously demonstrate that this is indeed the case, for mouse tumours such as the B16 melanoma or the N2a neuroblastoma. Both tumors express endogenous retroviruses normally silent, and by using RNA interference we demonstrated that knocking down these ERVs resulted in the rejection of the tumor cells, with re-expression of the sole ERV *env *gene reverting the effect. Adoptive transfer of regulatory T cells restored tumour progression of the knocked-down tumor cells, indicating that they are key to the ERV-mediated effect. These experiments strongly suggested that induction of ERVs, a process currently observed in tumors, including in humans, contributes to escape from immunosurveillance, via the immunosuppressive activity carried by the associated envelope proteins. Concerning the first hypothesis, on the impact of the immunosuppressive activity of the envelope protein of infectious retroviruses on viremia, recent experiments also indicate that this activity is absolutely required for productive infection in a mouse model, via inhibition of the host immune system.

## AS: Could this immunosuppressive activity play a role in the case of syncytins?

**TH**: A third hypothesis that we had not introduced in our 1998 PNAS paper concerns the syncytins: being *env *genes from endogenous retroviruses, they are expected to be not only fusogenic -in relation with syncytiotrophoblast formation- but also immunosuppressive. This actually proved to be the case, for at least one of them in both the primate and rodent lineage, leading to the working hypothesis that this property could play a role -or could have played a role in evolution- in the establishment of maternal-fetal tolerance, and even be associated with the emergence of placental mammals from egg-laying animals. Actually, such a hypothesis still remains a working model, and a series of experiments are underway in our laboratory using for instance knocked-in mice in which we have selectively abolished the immunosuppressive activity of the syncytins without altering their fusogenic activity, via specific mutations. Whatever the outcome of these experiments, it is already clear that endogenous retroviruses can no longer be considered simply as junk DNA, with their demonstrated involvement in a physiological process such as placentation.

## AS: So you would conclude that viruses harm us as much as they help us. What do you think of this idea?

**TH**: Your question could even be re-formulated in a more general way as follows: are mobile elements negative or positive? And the answer is they are both! Being insertional mutagens, mobile elements (and these include prokaryotic elements, retrotransposons, ERVs, etc.) are positive at the level of evolution, by generating diversity. In this respect it is remarkable that mobile elements are in general strongly repressed in the somatic cells, but repression is released to some extent in the germline, and this is true from Drosophila to mammals. And the germline is the right place for mutations to occur and generate variant offspring, which then will be subjected to Darwinian selection. But clearly mutations by insertion can be deleterious, and the best example is related to the insertional mutagenesis produced at the somatic cell level by simple oncogenic retroviruses, which can trigger tumors just in this way. And in Drosophila, the I retrotransposon can even induce embryonic lethality by excess retrotransposition. But of course mutations by insertion are not the sole possible effects of RVs and ERVs that indeed encode viral proteins which *per se *can have biological effects. These can be positive -the syncytin case- and they may be negative -the tumor case, via inhibition of immune surveillance.

## AS: Your group is mainly focusing on endogenous retroviruses. What are your future directions/thoughts?

**TH**: The refined study of ERVs taught us and will continue to teach us a lot on infectious retroviruses. This is expected because both are remarkably similar, which is consistent as they have common origins, but they have also a few differences which, if studied in detail and with perseverance, reveal important properties of both elements. An example of that arose in the course of our study of the primate and rodent syncytins. We found that all of them were indeed functional envelope proteins, that is, they were fusogenic, but we found that some of them were no longer immunosuppressive. This singularity actually allowed us to precisely map the domain responsible for this activity and to find mutations for specifically knocking-down this function without altering the « mechanical » property of the fusogenic envelope. This disjunction between the canonical fusion function of retroviral envelopes and the -often ignored- immunosuppressive function, then allowed us to show that the latter is indeed essential for viral replication and propagation in an immunocompetent host. And, finally, this was rendered possible via the analysis of ERV envelopes captured by ancestral hosts 40 My ago! So we are now investigating this function in detail, in relation with placenta formation for syncytins, inhibition of tumor immunosurveillance for endogenous retroviruses, and viremia for infectious retroviruses. We are also presently doing research on modified retroviral envelope proteins to tentatively derive « optimized » antigens, and have already obtained encouraging results on a veterinary vaccine.

## AS: How do you explain that only a few groups are interested in ERVs?

**TH**: I think that it is a question of fashion. And to be frank, it is much easier to get grants for HIV than for ERVs! Although I have to admit that my research has always been strongly supported by the CNRS, as well as by charity associations fighting against cancer, both of which I warmly thank. Also one has to acknowledge that if so much has been discovered on HIV, it is thanks to the very fundamental and in-depth virological studies that had been performed for so many years on ALV, MLV and other animal retroviruses; and I would like to add the ERVs to this list! In this respect, I think it is not useless to always recall that fundamental science is most probably the best way to be able in the long run to develop innovative therapeutics and vaccine strategies which will work, and that it has to be financially supported! But I think that most modern countries have now understood that research is essential for development, so I am confident that resolute individuals will always have the means to find their way.

## AS: What would you say to young students wanting to start out in research?

**TH**: Just do it! In fact it is a pity that a large fraction of the students are no longer interested in science. There are many possible reasons for that, maybe not enough science taught in school, insufficient popularization, and lack of social -and financial!- acknowledgment in our country. Many graduate students, and quite often the most gifted ones turn to business schools, and are lost to research. Maybe one positive outcome of the present financial crisis will be to re-direct students to science, but this will take time!

It is also our task to convince young people that scientific research is not so difficult, that science is one of the most complete forms of art; and if it requires a strong technical basis and intellectual rigor, it also leaves a great deal to intuition, promotes a sense of beauty and allows entry into the greatest terra incognita remaining to be explored by the new generations, all activities that are most probably deeply encoded in our genes of evolved primates!
